# Efficacy and Safety of “Bushen Huoxue Therapy”-Based Combined Chinese and Western Medicine Treatment for Diabetic Kidney Disease: an Updated Meta-Analysis of 2105 Patients

**DOI:** 10.1155/2022/3710074

**Published:** 2022-01-12

**Authors:** Hongdian Li, Shaoning Dong, Yashen Liu, Ni Tian, Wenxue Yang, Ao Dong, Na Li, Mianzhi Zhang

**Affiliations:** ^1^Beijing University of Chinese Medicine, Beijing 100029, China; ^2^Tianjin Academy of Traditional Chinese Medicine, Tianjin 300120, China; ^3^Dongfang Hospital of Beijing University of Chinese Medicine, Beijing 100700, China

## Abstract

**Background:**

Diabetic kidney disease (DKD) is the most important cause of the end-stage renal disease (ESRD) and the main cause of renal replacement therapy. Excessive inflammatory response and renal fibrosis are the keys to the development of this disease, and the conventional Western medical treatment is difficult to achieve and obtain long-term stable clinical results in all patients with DKD. Many studies have shown that Chinese medicine as a complementary and alternative medicine may be another therapeutic option to mitigate the progression of DKD to ESRD. In recent years, many doctors have used the Bushen Huoxue therapy to assist Western medicine in the treatment of the disease and have achieved certain clinical effects. However, most of the current studies are small sample studies, and there is no evidence-based confirmation.

**Objective:**

To systematically evaluate the efficacy and safety of the Bushen Huoxue therapy combined with conventional Western medicine in the treatment of DKD.

**Methods:**

A comprehensive search of literature databases such as CNKI, Wanfang, Pubmed, and Cochrane Library was conducted. The screening condition was that the control group was treated with conventional Western medicine and the experimental group was treated with Bushen Huoxue therapy's RCT on top of the control group, and the RCTs were published from January 2011 to October 2021. The Cochrane risk bias assessment tool was used for literature quality evaluation, and RevMan 5.3 software was used for statistical analysis.

**Results:**

A total of 23 RCTs were finally included, with a total of 2,105 patients. Meta-analysis results show that the experimental group can effectively improve the clinical efficacy (RR = 1.28, 95% CI (1.22, 1.34), *P* < 0.01), significantly reduce Crea (SMD = −0.45, 95% CI (−0.57, −0.33), *P* < 0.01), 24 h UTP (SMD = −0.57, 95% CI (−0.69, −0.45), *P* < 0.01), BUN (SMD = −0.36, 95%CI (−0.48, −0.24), *P* < 0.01), UAER (SMD = −1.58, 95% CI (−1.78, −1.37), *P* < 0.01), and blood sugar, and have certain medication safety (RR = 0.00, 95% CI (−0.03, 0.03), *P*=0.87).

**Conclusions:**

Chinese medicine based on the Bushen Huoxue therapy has a good clinical effect in the treatment of diabetic kidney disease and has certain safety. However, due to the limitation of the quality and quantity of the included literature, the above conclusion still needs more rational experiments to further verify.

## 1. Introduction

Diabetic kidney disease (DKD) is one of the most common microvascular complications of diabetes and a major cause of the end-stage renal disease (ESRD). Pooled data from 54 countries show that more than 80% of ESRD arises from diabetes, hypertension, or a combination of both and that ESRD is 10 times more prevalent in patients with diabetes than in those without diabetes [1]. As the incidence of diabetes increases, the population with DKD expands, and it is estimated that the number of people with DKD will increase by a factor of 1 by 2025 [2], and in developed countries, approximately 40% of people with DKD eventually face dialysis [3]. The socioeconomic and public health burden of DKD is significant, making the search for effective therapies to prevent and treat DKD critical.

The current treatment strategy for DKD aims to control blood glucose, blood pressure, and lipid levels by aggressive control, and although there are many Western drugs available for clinical treatment of DKD, only blocking renin-angiotensin-aldosterone system (RAAS) is an effective treatment, and commonly used drugs include angiotensin-converting enzyme inhibitors (ACEis), angiotensin receptor blockers (ARBs), and direct renin inhibitors (DRIs) [4–6], but these drugs are difficult to stop the inflammatory response and renal fibrosis [7]. In addition, recent studies have reported several noteworthy novel agents including sodium-glucose cotransporter 2 inhibitors (SGLT2is) that have beneficial effects in controlling the progression to DKD in diabetic patients. But these agents are still in early clinical experiments, and their efficacy and safety are not yet known. Therefore, researchers and clinicians are urgently searching for effective and safe drugs that can actually slow down the progression of DKD [8, 9]. Traditional Chinese medicine (TCM) has a history of thousands of years in treating kidney diseases, and DKD belongs to the categories of “edema,” “guangs,” and “turbidity of urine” in Chinese medicine. After decades of clinical observation and research, our team found that, according to the principles of TCM diagnosis, combined with its development process and clinical symptoms, this disease is a complex disease with kidney deficiency as the main cause [10]. In addition, the classical theory of TCM believes that prolonged illness is prone to blood stasis, and the long course of DKD leads to blood stasis, which must be the main pathological product of this disease. Therefore, Bushen Huoxue is the basic principle and important idea of treating this disease. In recent years, there have been more and more clinical experiments and systematic reviews on the treatment of DKD with herbs, but few studies have systematically evaluated the efficacy and safety of this therapy in combination with conventional Western medicine. Therefore, this study adopted an evidence-based medical approach to review and meta-analyze relevant clinical studies on the treatment of DKD using a combination of Western and herbal Chinese medicine based on the Bushen Huoxue therapy to evaluate the efficacy and safety of oral Bushen Huoxue herbs as an adjunctive treatment for DKD.

## 2. Information and Methods

### 2.1. Study Population

Patients who met the diagnosis of DKD and were staged using the Mogensen staging method.

### 2.2. Inclusion Criteria

(1) Type of study: an RCT experiment, whether blinded or not, without language restriction; (2) interventions: the control group was treated with conventional Western medicine for DKD, including blood glucose control, blood pressure lowering, lipid regulation, and other conventional treatments (no restriction on drug dose and dosage form); the experimental group added oral Chinese medicine preparation with Bushenhoxue as the main effect (no restriction on dosage and dosage form, including soup, granule, and pill, and the main effect of Chinese medicine preparation should be clearly mentioned in the original text as Bushenhoxue) on the basis of the control group; (3) outcome indicators: at least one of the following should be included: total clinical efficiency, 24-h urine protein quantification (24h UTP), blood creatinine (Crea), urea nitrogen (BUN), urinary albumin excretion rate (UAER), fasting blood glucose (FBG), and glycated hemoglobin (HbA1c); adverse reactions; and good balance and comparability between groups are the inclusion criteria.

### 2.3. Exclusion Criteria

Duplicate publications; literature with incomplete data or incomplete key information; included patients with other comorbidities affecting renal function; and interventions that included nonoral TCM treatments such as acupuncture, tui na, and proprietary Chinese medicine injections are the exclusion criteria.

### 2.4. Data Sources

**Figure 1:**
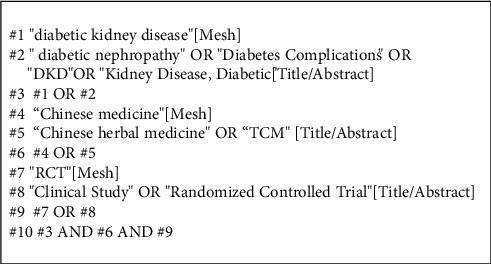
PubMed search strategy.

In this study, a comprehensive search was conducted for studies of biological therapeutic interventions for DKD in the last 10 years, with the search period specified as January 2011 to November 2021, and the databases searched included Chinese databases (China National Knowledge Infrastructure Factory (CNKI, https://www.cnki.net/), Wanfang Data Service Platform (https://www.wanfangdata.com.cn/index.html), VIP database (http://www.cqvip.com/), China Biomedical Literature Network (http://www.sinomed.ac.cn/)) and English databases (PubMed, Cochrane Library, Web of Science, and Springer databases). The search terms included “diabetic nephropathy,” “diabetic nephropathy,” “herbal medicine,” “Busenhoxue,” and “random.” The search was performed using subject terms + free words, and the search strategy is shown in Figure 1 for PubMed as an example. Journal literature from the library of the Beijing University of Chinese Medicine was also manually searched to supplement the search.

### 2.5. Data Extraction

A data extraction form was made, and two trained researchers extracted the data, and when there were differences of opinion, another researcher was added to discuss and solve the problem together. The original indexes of the relevant literature were verified and validated, and the original authors could be contacted by e-mail if there were any errors or ambiguous information, and if the original data could not be obtained indeed, the problematic literature was considered to be discarded.

### 2.6. Risk of Bias Evaluation

The risk and quality of the included literature were evaluated according to the risk of bias evaluation criteria recommended by the Cochrane Collaboration Network [11]. The assessment was made in terms of the method of random sequence generation, whether the personnel performing the assignment were strictly enforced, whether blinding was used, whether the outcome indicators were complete, whether positive results were selectively reported, and whether there were other possibilities of causing bias, respectively.

### 2.7. Statistical Methods

Meta-analysis was performed using the RevMan 5.3 software provided by the Cochrane Collaboration Network. Discontinuous variables were expressed as RR, and continuous variables were expressed as MD or SMD, and each effect size was expressed as a 95% confidence interval (CI). When *I*^*2*^ < 50%, it indicated that the studies were not heterogeneous and a fixed-effect model was used, and vice versa, it indicated that statistical heterogeneity existed, and subgroup analysis was performed to eliminate heterogeneity according to the possible heterogeneous factors. If statistical heterogeneity still existed, but clinical homogeneity was present, meta-analysis was performed using a random-effects model. If the heterogeneity was too large or clinically deemed inappropriate to combine, descriptive analysis was used. When the number of literature combining outcome indicators was >10, funnel plot analysis was used to analyze publication bias. Differences were considered statistically significant at *P* < 0.05.

## 3. Results

### 3.1. Literature Search Results and Basic Characteristics

**Figure 2:**
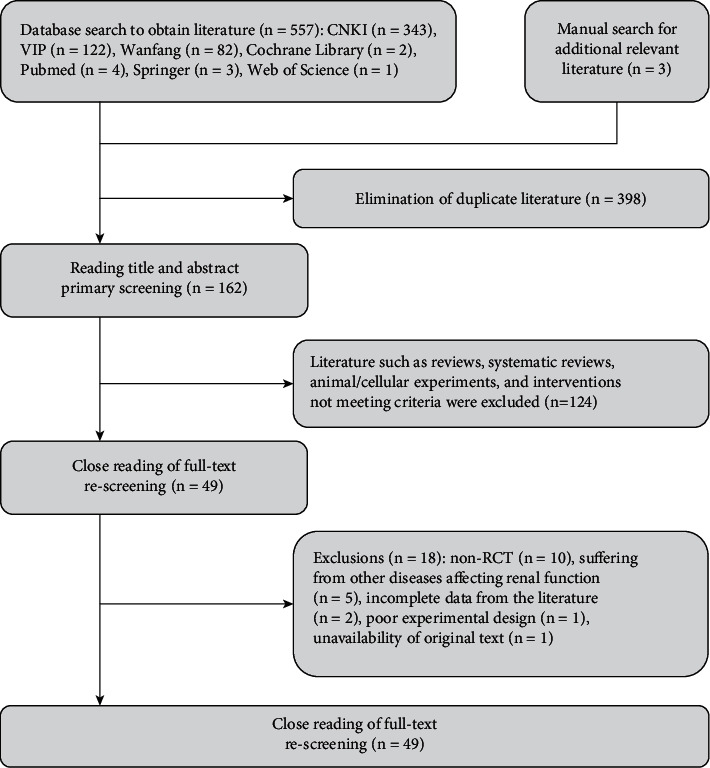
Literature screening process.

A total of 557 relevant studies were retrieved, and after screening, 23 RCTs with a total of 2,105 patients were finally included, with 1,053 patients in the experimental group and 1,052 patients in the control group, and all patients were matched at the baseline level. The sample size of an individual experiment ranged from 50 to 200 (see Figure 2 for the literature screening process and Table 1 for the literature characteristics).

### 3.2. Risk of Bias Evaluation Results

**Figure 3:**
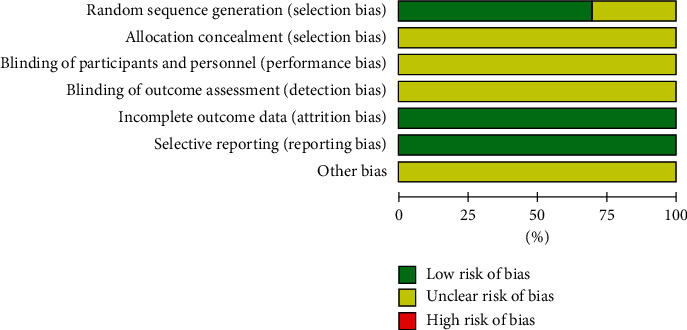
Risk of bias assessment graph for included RCTs.

**Figure 4:**
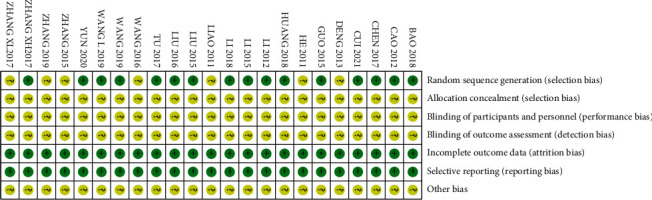
Distribution of risk of bias of included RCTs.

The quality of the included literature was evaluated using the “risk assessment tool” recommended by the Cochrane Collaboration: 16 of the 23 included studies [12–15, 17, 19, 20, 22–24, 26–28, 30, 33, 34] mentioned the specific randomization method used and therefore assessed as “low risk.” The other 7 [16, 18, 21, 25, 29, 31, 32] only mentioned the randomized grouping without mentioning the specific method used for allocation and were, therefore, evaluated as “unclear risk.” None of the included studies mentioned allocation concealment and blinding and were evaluated as “unclear risk.” All studies had clear outcome indicators and were evaluated as “low risk”; no duplicate publications or published biases were found in any of the studies and were evaluated as “low risk”; other biases were unknown and were evaluated as “unclear risk.” All data were completely reported and were comparable between groups (Figures 3 and 4).

### 3.3. Meta-Analysis Results

#### 3.3.1. Effect on Clinical Efficiency

**Figure 5:**
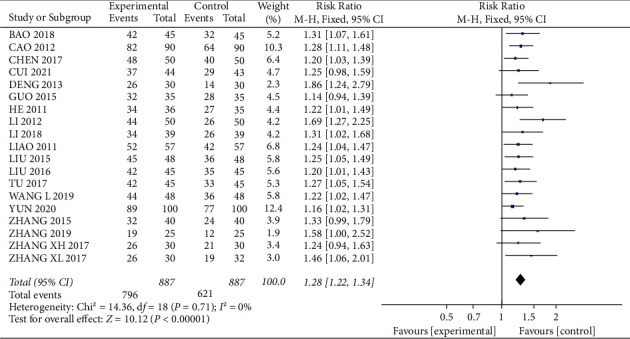
Forest plot comparing the overall response rate.

Total clinical effectiveness was mentioned in 19 of the 23 included studies [12–24, 27–32], with 887 patients in each of the experimental and control groups. There was no statistical heterogeneity between studies (*I*^2^ = 0%, *P*=0.71), and meta-analysis using a fixed-effects model showed that the clinical effective rate was higher in the experimental group than in the control group, with a statistically significant difference (RR = 1.28, 95% CI (1.22, 1.34), *P* < 0.01), indicating that Bushen Huoxue therapy adjuvant treatment of DKD can significantly improve the clinical efficacy (Figure 5).

#### 3.3.2. Effect on Crea

**Figure 6:**
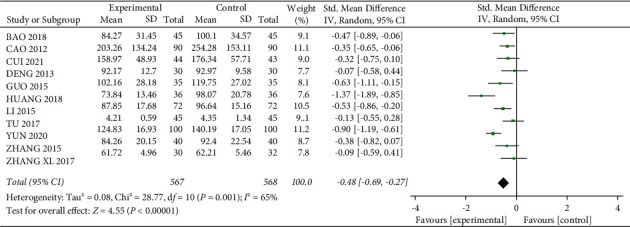
Forest plot comparing the Crea.

**Figure 7:**
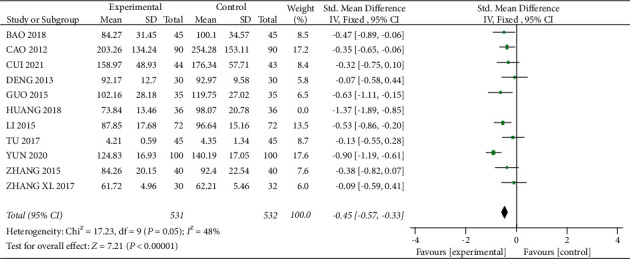
Forest plot for Crea comparison after sensitivity analysis.

A total of 11 studies [12, 13, 15–17, 24, 28, 29, 32–34] mentioned Crea, with 567 patients in the experimental group and 568 patients in the control group. The heterogeneity between studies was large (*I*^2^ = 65%, *P*=0.001), and because of the different assays used in each study, the SMD was used to express the results, and meta-analysis was performed using a random-effects model, which showed that the level of Crea was lower in the experimental group than in the control group after treatment, and the difference was statistically significant (SMD = −0.48, 95% CI (−0.69, −0.27), *P* < 0.01, Figure 6). After removing “Huang 2018,” the heterogeneity was reduced to 48% (Figure 7), and this experiment was considered as a main source of heterogeneity. The result showed a statistically significant difference (SMD = −0.45, 95% CI (−0.57, −0.33), *P* < 0.01), with a better reduction in Crea in the experimental group.

#### 3.3.3. Effect on 24 h UTP

**Figure 8:**
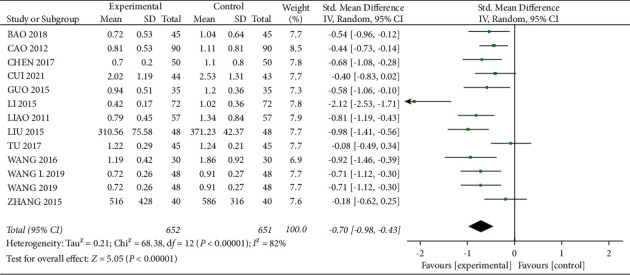
Forest plot comparing the 24 h UTP.

**Figure 9:**
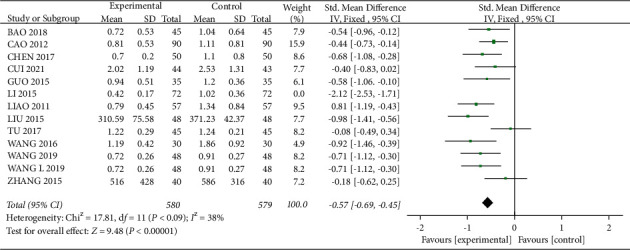
Forest plot for 24 h UTP comparison after sensitivity analysis.

A total of 12 studies [12–15, 21, 23–27, 29, 34] mentioned 24 h UTP, with 652 patients in the experimental group and 651 patients in the control group. There was statistical heterogeneity between studies (*I*^2^ = 82%, *P* < 0.01), and meta-analysis using a random-effects model showed that 24 h UTP levels were lower in the experimental group than in the control group, with a statistically significant difference (SMD = -0.70, 95% CI (-0.98, −0.43), *P* < 0.01, Figure 8). After removing “LI 2015,” the heterogeneity was reduced to 38% (Figure 9), which was considered as a main source of heterogeneity (SMD = -0.57, 95% CI (−0.69, −0.45), *P* < 0.01), which indicated that the experimental group was more effective in reducing 24 h UTP.

#### 3.3.4. Effect on UAER

**Figure 10:**
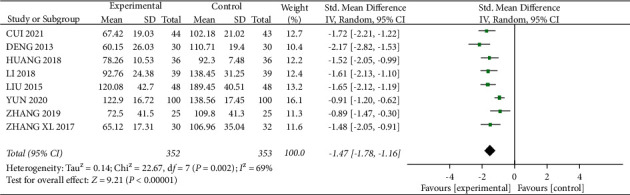
Forest plot comparing the UAER.

**Figure 11:**
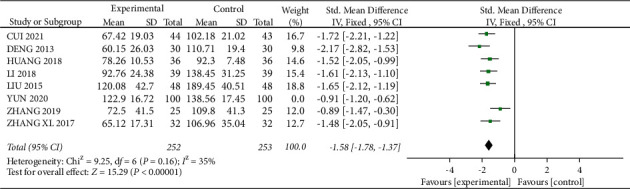
Forest plot for UAER comparison after sensitivity analysis.

A total of 8 studies [15, 16, 20, 23, 28, 31–33] mentioned UAER, with 352 patients in the experimental group and 353 patients in the control group. Statistical heterogeneity between studies was large (*I*^2^ = 69%, *P*=0.002), and meta-analysis using a random-effects model showed a statistically significant difference with SMD = −1.47, 95% CI (−1.78, −1.16), *P* < 0.01(Figure 10). Looking for sources of heterogeneity, heterogeneity was significantly reduced after removing “YUN 2020” (*I*^2^ = 35%, *P*=0.16, Figure 11), and the analysis was repeated using a fixed-effects model: SMD = −1.58, 95% CI (−1.78, −1.37), *P* < 0.01, and the experimental group reduced UAER, which was more effective.

#### 3.3.5. Effect on BUN

**Figure 12:**
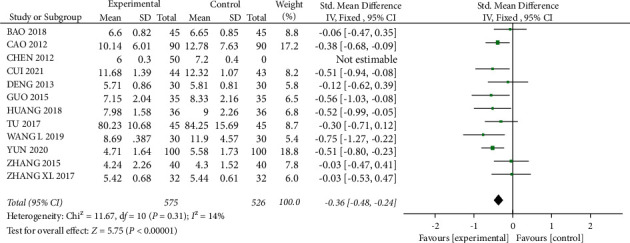
Forest plot comparing the BUN.

A total of 12 studies [12–17, 24, 26, 28, 29, 32, 33] mentioned BUN, with 575 patients in the experimental group and 526 patients in the control group. Statistical heterogeneity between studies was low (I^2^ = 14%, *P*=0.31), and meta-analysis using a fixed-effects model showed that BUN levels were lower in the test group than in the control group, with a statistically significant difference (SMD = −0.36, 95% CI (−0.48, −0.24), *P* < 0.01) (Figure 12).

#### 3.3.6. Effect on Glycemic Indexes

In this study, two blood glucose-related indicators were analyzed, including FBG and HbA1c, 13 studies recorded FBG and 6 studies recorded HbA1c, and meta-analysis was performed for both indicators, both expressed as MD, and according to the results of meta-analysis, the overall blood glucose indicators in the experimental group were lower than those in the control group, and the differences were statistically significant (Table 2, detailed forest plots are available in the Supplementary File).

#### 3.3.7. Adverse Reactions

**Figure 13:**
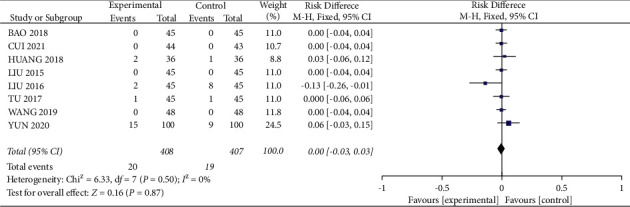
Forest plot comparing the adverse reactions.

Eight studies [12, 15, 22-24, 27, 28, 33] in the included literature mentioned adverse reactions, but only four of them [22,24,28,33] had patients with adverse reactions, and all patients in the other four studies did not have adverse reactions. A total of 20 patients in the experimental group had adverse reactions during treatment, including 5 cases of nausea and vomiting, 2 cases of headache with vertigo, 3 cases of loss of appetite, 6 cases of diarrhea, and 4 cases of fever; a total of 19 patients in the control group experienced adverse reactions, including 7 cases of nausea and vomiting, 6 cases of headache and vertigo, 2 cases of fever, 2 cases of loss of appetite, and 2 cases of diarrhea. Meta-analysis showed homogeneity between studies (*I*^2^ = 0%. *P*=0.50), and the differences were not statistically significant when analyzed using a fixed-effects model (RR = 0.00, 95% CI (−0.03, 0.03), *P*=0.87) (Figure 13), and the safety of medication administration was comparable in the experimental and control groups.

### 3.4. Publication Bias

**Figure 14:**
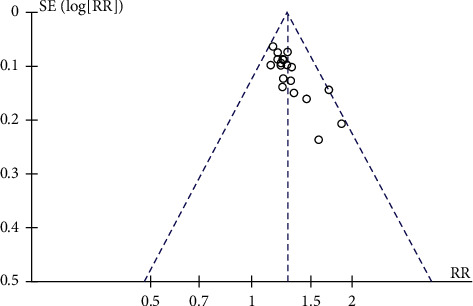
Overall response rate's publication bias funnel chart.

**Figure 15:**
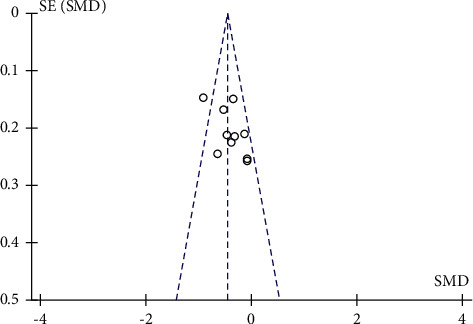
Crea's publication bias funnel chart.

**Figure 16:**
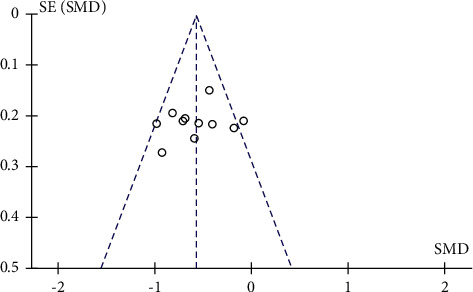
24 h UTP's publication bias funnel chart.

**Figure 17:**
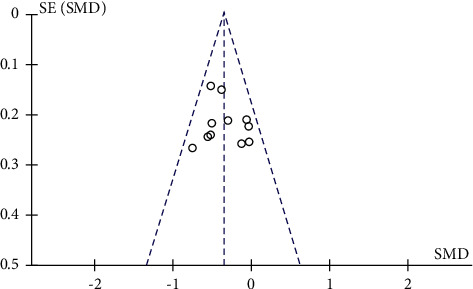
BUN's publication bias funnel chart.

**Figure 18:**
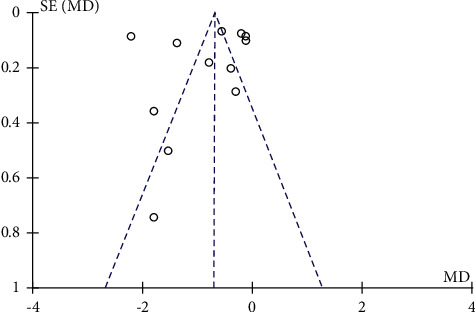
FBG's publication bias funnel chart.

Funnel plots were plotted for studies with >10 literature on combined outcome indicators, and total effective rate, Crea, 24h UTP, BUN, and FBG after treatment showed significant asymmetry in the funnel plots (Figures 14–18), indicating publication bias in the included studies.

## 4. Discussion

This study completed a systematic evaluation in accordance with the Cochrane risk bias assessment tool version 5.1.0 and the PRISMA statement [35]. The results of the study showed that Bushen Huoxue therapy adjuvant to Western medicine for DKD significantly reduced indicators of renal damage, improved overall clinical efficiency, and reduced blood glucose; in terms of medication safety, Bushen Huoxue therapy had no significant side effects, and this result was consistent with the results of this study's single largest sample size literature Yun 2020 [28] (*n* = 200).

DKD is part of systemic microvascular disease and glomerulosclerosis caused by diabetes mellitus, which is the leading cause of renal replacement therapy in Europe and the United States, accounting for about 1/2 of cases, and it is the second most common cause of ESRD after glomerular disease in China [1]. DKD more rapidly progresses to ESRD than nondiabetic-caused CKD, and therefore, there is an urgent need to find effective preventive measures to delay the onset of DKD [36]. In recent times, Chinese herbal medicine has been used as a complementary and necessary combination drug treatment for renal disease in many patients in China due to its less adverse effects and more effective interventions, and a study from Taiwan, China, showed that the use of Chinese herbal medicine in CKD patients significantly reduced the risk of developing ESDR by approximately 60% [37]. In addition to clinical efficacy, the safety of herbal medicine in the treatment of this disease is of particular concern, and many attempts have been made to find out whether herbal medicine has a protective effect on the kidney, although most clinical experiments have shown [38–40] that the safety of herbal medicine in the adjuvant treatment of DKD does not have a significant advantage over the effect of using Western drugs alone, but the protective effect of herbal medicine on the kidney has been initially verified in some animal experiments, and the antioxidant and anti-inflammatory effects of TCM were considered to be the basis of its protective effect [41].

The key to Bushen Huoxue therapy of this disease is to grasp its fundamental pathogenesis. Diabetes mellitus over time consumes yin and injures qi and accumulates heat and injures fluid, the kidneys are involved, kidney qi is depleted, blood stasis is prolonged, and the peripheral veins and viscera are not allowed to flow, so the kidney function is completely lost in the advanced stage. The disease is characterized by a combination of deficiency and actuality. The disease is characterized by a mixture of deficiency and reality, and the deficiency evidence is more obvious. The flexible addition and reduction in medicine based on the principle of holistic view and evidence-based treatment are the outstanding advantages of traditional Chinese medicine.

## 5. Limitations

Despite our working group's best efforts to control for literature inclusion criteria, literature quality, and other details, there are still certain shortcomings in this study that deserve to be explored for future avoidance and improvement. First, it was affected by the quality of the literature. None of the literature included in this study mentioned blinding and allocation concealment, and therefore, the original literature outcomes are unknown for selective bias, implementation bias, and measurement bias. However, it is reassuring to note that all the literature is RCT, with no omission of endings, which ensures that the results of this study are more credible. Second, a total of 23 studies were included in this study, and the study period was limited to 10 years, which resulted in an inadequate amount of literature. Our reflection on this point is that Chinese clinical trials in China in the last decade have been relatively standardized, and we made a selection to ensure the quality of the study and strictly limited the time. In addition, our results showed no difference in safety between combined Chinese and Western medicine treatment and Western medicine treatment alone, which may be related to the lack of strict control of drug dose and duration in this study, and to the number of literature. Finally, the funnel plot showed publication bias in this study, which may be due to the fact that negative results are difficult to publish and positive results are more likely to be reported. To address the above issues, in future studies, we will continue to conduct large-sample, multicenter, rigorous clinical trials and high-quality evidence-based analyses to verify the accuracy of the findings of this study.[42]

## Figures and Tables

**Table 1:** Basic characteristics of included RCTs.

Study	Type of Experiment	Sample size	Random method	Interventions		Period of treatment	Outcomes	Adverse reactions
		C/E		C	E			C/E
Bao [12]	RCT	45/45	Lottery	*N*	*N* + Bushen Huoxue granules	12 weeks	①②③④⑥⑦	0/0
Cao and Shao [13]	RCT	90/90	Lottery	*N*	*N* + Bushen Huoxue decoction	12 weeks	①②③④⑥	—
Chen and Lu [14]	RCT	50/50	Lottery	*N*	*N* + Baoshentongluo decoction	8 weeks	①②④⑥	
Cui et al. [15]	RCT	43/44	TRD	*N*	*N* + Baoshentongluo decoction	12 weeks	①③④⑤⑥⑦	0/0
Deng et al. [16]	RCT	30/30	—	*N*	*N* + Jiangtanghuoxue decoction	12 weeks	①②③⑤⑥	—
Guo and Ma [17]	RCT	35/35	TRD	*N*	*N* + Bushen Huoxue decoction	12 weeks	①②③④⑥	—
He [18]	RCT	35/37	—	*N*	*N* + Yiqihuoxuebushen decoction	12 weeks	①	—
Li [19]	RCT	50/50	Lottery	*N*	*N* + Bushen Huoxuexingyu decoction	4—8 weeks	①	—
Li [20]	RCT	39/39	TRD	*N*	*N* + Bushen Huoxue decoction	8 weeks	①⑤	—
Liao [21]	RCT	57/57	—	*N*	*N* + Bushen Huoxue decoction	8weeks	①②④	—
Liu et al. [22]	RCT	45/45	Lottery	*N*	*N* + Bushentongluoxingyu decoction	12 weeks	①⑦	8/2
Liu [23]	RCT	45/45	Lottery	*N*	*N* + Yiqihuoxuebushen decoction	8–12 weeks	①②④⑤⑦	0/0
Tu [24]	RCT	45/45	TRD	*N*	*N* + Yishenxiaoke decoction	12 weeks	①③④⑥⑦	1/1
Wang [25]	RCT	30/30	—	*N*	*N* + Bushen Huoxue decoction	8 weeks	①②④	—
Wang and He [26]	RCT	30/30	TRD	*N*	*N* + Bushen Huoxuexiezhuo decoction	4weeks	①③⑤⑥	—
Wang [27]	RCT	48/48	TRD	*N*	*N* + Jinguishenqi pills combined with Taohongsiwu decoction	8weeks	①②④⑦	0/0
Yun et al. [28]	RCT	100/100	Lottery	*N*	*N* + Baoshentongluo decoction	8 weeks	①③⑤⑥⑦	9/15
Zhang and Zhi [29]	RCT	40/40	—	*N*	*N* + Jiangtanghuoxue decoction	4weeks	①②③④⑥	—
Zhang [30]	RCT	30/30	TRD	*N*	*N* + Xinshenkang capsules	24 weeks	①	—
Zhang [31]	RCT	25/25	—	*N*	*N* + Bushen Huoxue decoction	12 weeks	①⑤	—
Zhang et al. [32]	RCT	32/30	—	*N*	*N* + Bushen Huoxue decoction	8 weeks	①②③⑤⑥	—
Huang [33]	RCT	36/36	TRD	*N*	*N* + Bushen Huoxue decoction	12 weeks	①②③④⑤⑥⑦	1/2
Li and Ren [34]	RCT	72/72	TRD	*N*	*N* + Bushen Huoxue decoction	8 weeks	②③④	—

TRD: table of random digit; N: conventional Western medicine for DKD, including blood glucose control, blood pressure lowering, lipid regulation, and other conventional treatments; E: experimental group; C: control group; -: not mentioned; ① total effective rate; ② glucose-related indexes (including at least one of FBG and HbA1c); ③ Crea; ④ 24h UTP; ⑤ UAER; ⑥ BUN; and ⑦ adverse effects. Details of group E interventions are as follows: Bushen Huoxue decoction/granules [12, 13, 17, 20, 21, 25, 31–34] is a herbal preparation with very clear Bushen Huoxue effects only. In addition, Baoshentongluo decoction [14, 15, 28], Yishenxiaoke decoction [24], Jiangtanghuoxue decoction [16, 29], Yiqihuoxuebushen decoction [18, 23], Bushentongluoxingyu decoction [19, 22], Bushen Huoxuexiezhuo decoction [26], Jinguishenqi pills combined with Taohongsiwu decoction [27], and Xinshenkang capsules [30] all have Bushen Huoxue as the main effect Chinese herbal formulas, and these interventions and specific medications are clearly described in the corresponding original texts.

**Table 2:** Meta-analysis results of blood glucose-related indicators.

Outcomes	Number of included studies	Meta-analysis results		Effect model	Heterogeneity test	
		MD (95%CI)	*P* value		*P* value	*I* ^2^ (%)
FBG	13 [12-17, 21, 23, 27, 29, 32–34]	−0.86 (−1.30, 0.43)	<0.01	Random	<0.01	97
HbA1c	6 [16, 23, 29, 32–34]	−0.38 (−0.71, −0.06)	<0.01	Random	0.02	92

## Data Availability

Extracted data used to support the results of this study are available from the corresponding author upon request.

## Supplementary Materials

Supplementary Materials Figure 1: forest plot comparing the FBG. Figure 2: forest plot comparing the HbA1c.

## References

[1] United States Renal Data System (2014). International comparisons. *United States Renal Data System. 2014 USRDS Annual Data Report: Epidemiology of Kidney Disease in the United States*.

[2] White S. L., Chadban S., Report K. (2014). Kidneys in Diabetes: temporal trends in the epidemiology of diabetic kidney disease and the associated health care burden in Australia. *Report of the Kidney in Diabetes*.

[3] Ahmad J. (2015). Management of diabetic nephropathy: recent progress and future perspective. *Diabetes & Metabolic Syndrome: Clinical Research Reviews*.

[4] Li R., Bilik (2013). Medical costs associated with type 2 diabetes complications and comorbidities. *American Journal of Managed Care*.

[5] American Diabetes Association (2018). Glycemic targets: standards of medical care in diabetes—2018. *Diabetes Care*.

[6] Jia W., Weng J., Zhu D. (2019). Standards of medical care for type 2 diabetes in China 2019. *Diabetes/Metabolism Research and Reviews*.

[7] National Kidney Foundation (2012). KDOQI clinical practice guideline for diabetes and CKD: 2012 update. *American Journal of Kidney Diseases*.

[8] Alicic R. Z., Johnson E. J., Tuttle K. R. (2018). SGLT2 inhibition for the prevention and treatment of diabetic kidney disease: a review[J]. *American Journal of Kidney Diseases*.

[9] Pecoits-Filho R., Perkovic V. (2017). Are SGL2 inhibitors ready for prime time for CKD?. *Clinical Journal of the American Society of Nephrology*.

[10] Zhang M. Z., Zhang D. N. (2016). Clinical experience of Zhang Daning in the treatment of diabetic nephropathy[J]. *Chinese Journal of Traditional Chinese Medicine*.

[11] Deeks J., Higgins J., Altman D. (2011). Chapter 9-analysing Dataand Undertaking meta-analyses: cochrane Handbook for Systematic Reviews of Interventions Version 5.1. *Cochrane Handbook for Systematic Reviews of Interventions*.

[12] Bao X. (2018). Clinical study of the effect of the method of Bushen Huoxue on the level of CTGF in urine of diabetic nephropathy. *Sichuan Traditional Chinese Medicine*.

[13] Cao H., Shao Z. (2012). Observation on the curative effect of the method of Bushen Huoxue in the treatment of diabetic nephropathy. *Hubei Journal of Traditional Chinese Medicine*.

[14] Chen X., Lu X. (2017). The therapeutic effect of Bushen Huoxue combined with western medicine on patients with early diabetic nephropathy and its influence on related inflammatory factors. *Shaanxi Journal of Traditional Chinese Medicine*.

[15] Cui F., Zhao W., Wang Y. (2021). Clinical study on 44 cases of diabetic nephropathy with deficiency of both qi and yin and blood stasis with “Baoshentongluo” combined with basic treatment program intervention. *Jiangsu Traditional Chinese Medicine*.

[16] Deng S., Fan P., Zhang Z. (2013). The clinical efficacy of Yishenhuoxue combined with irbesartan in the treatment of early diabetic nephropathy. *Chinese Journal of Gerontology*.

[17] Guo C., Ma X. (2015). Observation on the therapeutic effect of the method of Bushen Huoxue in the treatment of diabetic nephropathy. *Asia-Pacific Traditional Medicine*.

[18] He J. (2011). Treating 37 cases of diabetic nephropathy with Yiqihuoxuebushen Decoction combined with Irbesartan. *Shaanxi Journal of Traditional Chinese Medicine*.

[19] Li B. (2012). Therapeutic effect analysis on 100 cases of early diabetic nephropathy treated with the method of Bushen Huoxuehuayu. *Journal of Chinese and Foreign Medical Research*.

[20] Li H. (2018). Clinical study of Danqibushenhuoxue Decoction combined with Benazepril in the treatment of early diabetic nephropathy. *Practical Clinical Journal of Integrated Traditional Chinese and Western Medicine*.

[21] Liao J. (2011). Yishenhuoxue Decoction in the treatment of 57 cases of early diabetic nephropathy. *Clinical Medical Engineering*.

[22] Liu D., Wu C., Gong Z. (2016). Discussion on the clinical efficacy of Bushentongluoxingyu therapy in the treatment of diabetic nephropathy. *Digest of World Latest Medical Information (Continuous Electronic Journal)*.

[23] Liu P. (2015). Observation on the curative effect of drugs for yiqibushenhuoxue in the treatment of diabetic nephropathy. *Beifang Pharmacy*.

[24] Tu Y. (2017). Clinical efficacy of conventional western medicine combined with Yishenxiaoke decoction in the treatment of Shenxuxueyu-type early diabetic nephropathy. *Journal of Clinical Rational Use*.

[25] Wang M. (2016). Observation on the curative effect of Bushen Huoxue Therapy in the treatment of diabetic nephropathy. *New World of Diabetes*.

[26] Wang L., He Z. (2019). Effect of Bushen Huoxuexiezhuo decoction on renal function and serum inflammatory factors in patients with type 2 diabetic nephropathy and renal failure. *Chinese Emergency in Traditional Chinese Medicine*.

[27] Wang X. (2019). Observation on the clinical efficacy of Jinguishenqi pill combined with Thongsiwu decoction in patients with early diabetic nephropathy. *China Medical Journal of Metallurgical Industry*.

[28] Yun R., Zhang S., Sun L. (2020). Baoshentongluo decoction and valsartan capsules in the treatment of diabetic nephropathy clinical study. *Journal of Changchun University of Traditional Chinese Medicine*.

[29] Zhang W., Zhi Y. (2015). Observation on the curative effect of Jiangtang Yishen Huoxue Decoction combined with Alprostadil in the treatment of diabetic nephropathy. *China Journal of Traditional Chinese Medicine Science and Technology*.

[30] Zhang X. (2017). A preliminary study of Bushen Huoxue method on the influencing factors of renal tubular injury in diabetic nephropathy. *Journal of Traditional Chinese Medicine Research*.

[31] Zhang H. (2019). Analysis of the effect of traditional Chinese medicine Huangqibushenhuoxue decoction combined with western medicine in the treatment of diabetic nephropathy. *Electronic Journal of Integrated Traditional Chinese and Western Medicine Cardiovascular Diseases*.

[32] Zhang X., Jin F., Ding W. (2017). Bushen Huoxue prescription combined with valsartan capsules in the treatment of 30 cases of stage III diabetic nephropathy. *Western Journal of Traditional Chinese Medicine*.

[33] Huang Y. (2018). *Bushen Huoxue’s Clinical Study on Oxidative Stress in Early Diabetic Nephropathy*.

[34] Li W., Ren W. (2015). Analysis of the clinical efficacy of the method of invigorating the kidney and promoting blood circulation combined with enalapril on diabetic nephropathy. *Chinese Journal of Basic Medicine in Traditional Chinese Medicine*.

[35] Moher D., Liberati A., Tetzlaff J. (2009). Preferred reporting items for systematic reviews and meta-analyses: the PRISMA statement. *BMJ*.

[36] Piccoli G. B., Grassi G., Cabiddu G. (2015). Diabetic kidney disease: a syndrome rather than a single disease. *The Review of Diabetic Studies*.

[37] Lin M.-Y., Chiu Y.-W., Chang J.-S. (2015). Association of prescribed Chinese herbal medicine use with risk of end-stage renal disease in patients with chronic kidney disease. *Kidney International*.

[38] Sheng X., Dong Y., Cheng D., Wang N., Guo Y. (2020). Efficacy and safety of Bailing capsules in the treatment of type 2 diabetic nephropathy: a meta-analysis. *Annals of Palliative Medicine*.

[39] Yu Z., Zhang W., Li B. (2021). Efficacy and safety of acupuncture combined with Chinese Herbal Medicine for diabetic nephropathy. *Medicine*.

[40] Liu Y. Y., Guo Z. A., Zhou T. Y. (2021). Clinical efficacy and safety evaluation of Yiqiyangyintongluo Decoction for diabetic nephropathy G3aA2 stage Qiyinliangxuxueyu evidence. *Chinese Journal of Traditional Chinese Medicine*.

[41] Yang X., Hu C., Wang S., Chen Q. (2020). Clinical efficacy and safety of Chinese herbal medicine for the treatment of patients with early diabetic nephropathy. *Medicine*.

[42] Liao J. (2011). Effect of yishen huoxue decoction on 57 cases of early diabetic nephropathy. *Clinical Medical Engineering*.

