# Supplementary material for: Efficacy and Safety of “Bushen Huoxue Therapy”-Based Combined Chinese and Western Medicine Treatment for Diabetic Kidney Disease: an Updated Meta-Analysis of 2105 Patients
Source: Evid Based Complement Alternat Med. 2022 Jan 12;2022:3710074. doi: 10.1155/2022/3710074 (PMC8769826; doi:10.1155/2022/3710074)
Supplement: Supplementary Materials — Figure 1: forest plot comparing the FBG. Figure 2: forest plot comparing the HbA1c. [file 3710074.f1.docx]

**Supplementary file : effect on glycemic indexes**


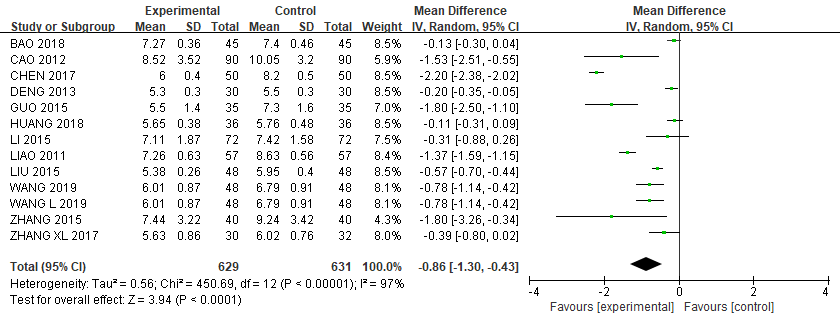


Fig. 1 Forest plot comparing the FBG


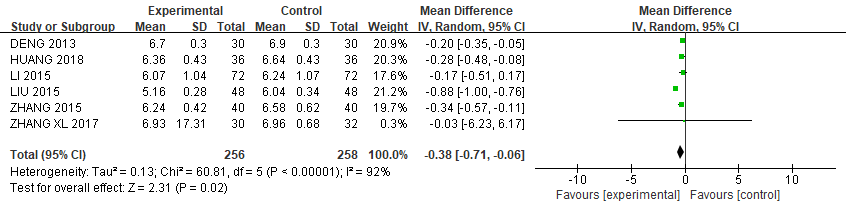


Fig. 2 Forest plot comparing the HbA1c
